# Potential of Recycling Cauliflower and Romanesco Wastes in Ruminant Feeding: *In Vitro* Studies

**DOI:** 10.3390/ani10081247

**Published:** 2020-07-22

**Authors:** Trinidad de Evan, Andrea Vintimilla, Eduarda Molina-Alcaide, María José Ranilla, María Dolores Carro

**Affiliations:** 1 Departamento de Producción Agraria, Escuela Técnica Superior de Ingeniería Agronómica, Agroalimentaria y de Biosistemas, Universidad Politécnica de Madrid, Ciudad Universitaria, 28040 Madrid, Spain; t.deevan@alumnos.upm.es (T.d.E.); andre_v2105@hotmail.com (A.V.); 2 Estación Experimental del Zaidin (Consejo Superior de Investigaciones Científicas), Profesor Albareda 1, 18008 Granada, Spain; molina@eez.csic.es; 3 Departamento de Producción Animal, Universidad de León, 24071 León, Spain; mjrang@unileon.es; 4 IGM (CSIC-ULE), Finca Marzanas s/n, 24346 Grulleros, León, Spain

**Keywords:** cauliflower, Romanesco, wastes, *in vitro* rumen fermentation, *in vitro* intestinal digestibility, CH_4_

## Abstract

**Simple Summary:**

The use of vegetable wastes in animal feeding is increasing worldwide, but the knowledge of the nutritional value of some of them is still limited. In this study, the nutritive value for ruminants of different fractions of cauliflower and Romanesco wastes (leaves, stems and sprouts) was assessed using *in vitro* techniques. In addition, we analyzed the effect of increasing the substitution rate of cauliflower for commercial concentrate in a dairy sheep diet containing 40% of alfalfa hay and 60% of concentrate. All fractions of both vegetables had high amounts of water, but their dry matter was rich in protein and sugar and it was extensively fermented *in vitro* by ruminal microorganisms. Stems and sprouts were more rapidly fermented than leaves, but there were only minor differences between the two assessed vegetables. In an *in vitro* study with diets for dairy sheep with concentrates containing increased amounts of dried cauliflower, we observed that cauliflower can be included up to 24% of the concentrate without any negative effect on rumen fermentation. The results indicate that cauliflower and Romanesco wastes could be used in ruminant feeding, but *in vivo* trials are needed to confirm the *in vitro* results.

**Abstract:**

The nutritive values for ruminants of cauliflower (CAU) and Romanesco (ROM) wastes (leaves, stems and sprouts) were assessed by analyzing their chemical composition, *in vitro* ruminal fermentation, and *in vitro* intestinal digestibility. In addition, the *in vitro* ruminal fermentation of diets containing increasing amounts of CAU was studied. The dry matter (DM) content of leaves, stems and sprouts of both vegetables was lower than 10%, but they contained high crude protein (CP; 19.9 to 33.0%) and sugar (16.3 to 28.7%) levels, and low neutral detergent fiber (21.6 to 32.3%). Stems and sprouts were more rapidly and extensively fermented in the rumen than leaves, but there were only minor differences the fermentation profiles of both vegetables. The estimated metabolizable energy content ranged from 9.3 (leaves) to 10.8 (sprouts) MJ/kg DM. The CP rumen degradability (12-h *in situ* incubations) was greater than 80.0% for all fractions, and the *in vitro* intestinal digestibility of CP ranged from 85.7 to 93.2%. The inclusion of up to 24% of dried CAU in the concentrate of a mixed diet (40:60 alfalfa hay:concentrate) increased the *in vitro* rumen fermentation of the CAU diet, but did not affect methane (CH_4_) production, indicating the lack of antimethanogenic compounds in CAU.

## 1. Introduction

Consumers are progressively aware of the importance of a healthy diet to reduce the incidence of numerous dietary illnesses, such as obesity, type 2 diabetes and heart disease, among others [1]. There is a consensus that a healthy diet must include five portions a day of vegetables and fruits, but average daily consumption is lower in most populations across the world [2]. In order to increase the intake of vegetables, new forms of packaging and presentation have been introduced in the market, such as frozen vegetables, ready-to-eat salads, bagged and canned vegetables, etc. The marketing of these products often results in high amounts of vegetable wastes because some parts of the vegetables, or even the whole vegetables, are discarded for not meeting the required quality standards. Vegetable wastes could be used in animal feeding, but their high-moisture content is an important limitation for their preservation. However, some low-cost drying options (i.e., solar tunnel dryers [3]) are being implemented in some countries, and other preservation options, such as ensiling, are being used in the practice for different vegetable wastes [4,5]. The utilization of vegetable wastes in livestock feeding may have multiple benefits, such as reducing feeding costs, direct competition for human-edible feeds, and the environmental problems caused by waste accumulation [6]. In addition, vegetable wastes may contain bioactive compounds that can improve the quality of milk and meat and decrease CH_4_ emissions from ruminants [7].

Previous studies [8,9,10,11] have analyzed the chemical composition and nutritive value of whole cauliflower (CAU; Brassica oleracea var. botrytis) and CAU leaves, indicating that they are a good source of protein and sugar. However, there is a lack of information on nutritive value of other CAU fractions (stems and sprouts). Some studies have reported a high intake of diets containing CAU leaves in goats [8,12], bulls [13], pigs [14] and rabbits [15] and analyzed their effects on animal performance and product quality. However, little is known on the digestive utilization of CAU fractions in ruminants. In addition, to our best knowledge, there is no information on the nutritive value for ruminants of Romanesco (ROM; *Brassica oleracea* var. botrytis cultivar group), another Brassica vegetable whose production and demand has sharply increased in the last few years [16]. 

Both CAU and ROM contain numerous bioactive compounds [17] that might modulate ruminal fermentation and reduce CH_4_ emissions, as it has been observed for other vegetables [7,18,19], but to our best knowledge there is no information on this point. The aim of this study was therefore to assess the potential nutritive values for ruminants of different fractions (leaves, stems and sprouts) of CAU and ROM by analyzing their chemical composition, *in vitro* ruminal fermentation and *in vitro* intestinal digestibility. As a second objective, the possibility of replacing conventional feeds with increasing amounts of CAU in ruminant diets was investigated by measuring the *in vitro* fermentation parameters of the diets and CH_4_ production.

## 2. Materials and Methods

### 2.1. Sheep and Feeding

Four mature Lacaune sheep (63.1 ± 2.05 kg body weight), each provided with a permanent rumen cannula, were used as donors of rumen fluid for the *in vitro* incubations and for conducting the *in situ* incubations necessary to measure the *in vitro* intestinal digestibility. Housing and feeding conditions have been described by de Evan et al. [20]. The sheep were cared and managed according to the Spanish regulations for experimental animal protection. Procedures for rumen contents collection were approved by the Institutional Animal Care and Use Committee of the Comunidad Autónoma de Madrid (PROEX 035/17).

### 2.2. Cauliflower and Romanesco Wastes 

Samples of CAU and ROM (about 10 kg) were obtained from local markets on three different weeks between October and December 2017. Samples of each vegetable obtained the same week were pooled, weighed, and separated into three fractions: leaves, stems and sprouts. Each fraction was weighed, cut into pieces and dried at 40 °C until constant weight. Samples were then ground to pass a 2 mm sieve, and a subsample was further ground (1 mm size) for chemical analyses and *in vitro* incubations.

### 2.3. Experiment 1. In Vitro Ruminal Fermentation and Intestinal Digestibility of Cauliflower and Romanesco Wastes

The *in vitro* rumen fermentation of each vegetable fraction was assessed in two different incubation trials performed according the Goering and Van Soest procedure [21] as detailed by de Evan et al. [20]. The objective of the first incubation was to determine the gas production kinetics of the samples, whereas the main fermentative parameters were assessed in the second one. In both incubations, contents of the rumen of each sheep were obtained before feeding, filtered through four layers of cheesecloth, and transported to the laboratory into thermal flasks. The rumen fluid was mixed with the Goering and Van Soest [21] culture medium in a 1:4 proportion. The procedure was conducted at 39 °C under CO_2_ flushing. No trypticase and NH_4_HCO_3_ was used in the culture medium to avoid adding exogenous nitrogen. Samples of each vegetable fraction (200 mg DM) were carefully weighted into 60-mL vials and 20 mL of the rumen fluid and culture medium mixture were added using a peristaltic pump (Watson-Marlow 520UIP31; Watson-Marlow Fluid Technology Group, Cornwall, UK). Vials were sealed with rubber stoppers and incubated at 39 °C for 144 and 24 h in the first and second incubation trial, respectively. In the first incubation trial, a pressure transducer (Delta Ohm DTP704-2BGI, Herter Instruments SL, Barcelona, Spain) and a plastic syringe were used to measure the gas production at 3, 6, 9, 12, 15, 22, 26, 31, 36, 48, 58, 72, 96, 120 and 144 h. In addition, the potential *in vitro* degradability of DM (PDDM) was determined by measuring the DM disappearance after the incubation of samples (300 mg DM) weighted into Ankom Corp #57 bags (30 µm pore size; Ankom Technology Corp., Fairport, NY, USA) in buffered ruminal fluid at 39 °C for 144 h in an Ankom Daisy II (Ankom Technology, Fairport, NY, USA) incubator, as described by de Evan et al. [20]. A mixture of ruminal fluid from the four sheep and the culture medium previously described was used. After 144 h, bags were washed with cold water, dried at 60 °C for 48 h, and weighed to calculate PDDM. In addition, one sample of each sugar beet pulp and wheat distiller’s dried grains with solubles (DDGS) were included in the incubation to be a reference for the estimation of metabolizable energy (ME) content, as described below. Data on *in vitro* fermentation of both feeds have been already published by de Evan et al. [20]. 

In the second *in vitro* incubation, gas production and the pH of the vials content were measured after 24 h as described before. Vials were then uncapped, their content was homogenized before measuring the pH, and 3 mL of vials content were mixed with 3 mL of 0.5 M HCl and frozen (−20 °C) until volatile fatty acid (VFA) and NH_3_-N analyses. In both incubations, two blanks (vials without substrate) per inoculum were included to measure the endogenous gas production. Four replicates per sample were obtained by using the ruminal fluid from each sheep independently as inoculum. 

The *in vitro* DM and CP intestinal digestibility were determined following the procedure of Gargallo et al. [22] as adapted by de Evan et al. [20]. Two g of each sample (2 mm ground) were weighed in duplicate into nylon bags (5 × 15 cm; 46 μm pore size), which were incubated in the rumen of each sheep immediately before the morning feeding for 12 h. After withdrawal, bags were rinsed to eliminate feed particles and immediately frozen (−20 °C) for one week. Finally, bags were thawed, washed with cold water in a turbine washing machine (3 cycles of 5 min each) and frozen before being lyophilized and weighed. The incubation residues were analyzed for N content and the *in situ* DM and CP degradability after 12 h incubation was calculated. Residues of 12 h incubation were pooled by vegetable fraction, and 0.3 g of each were carefully weighed in duplicate into Ankom R510 bags cut in half (50 µm pore size; 5 × 5 cm). Bags were incubated at 39 °C in a 0.1 N HCl solution (pH 1.9) containing 1 g/L of pepsin (P-7000, Sigma, St. Louis, MO, USA) for 1 h in the Daisy^II^ incubator, and then were washed under running tap water. Bags were further incubated at 39 °C for 24 h in a 0.5 M KH_2_PO_4_ buffer (pH 7.75) containing 3 g/L of pancreatin (P-7545, Sigma, St. Louis, MO, USA) and 50 ppm of thymol, and then washed in a turbine washing machine (cold water, 2 cycles, 5 min), dried (40 °C, 72 h), and weighed. Incubation residues were pooled by sample and sheep and their N concentration were analyzed to calculate the *in vitro* CP intestinal digestibility. 

### 2.4. Experiment 2. In Vitro Fermentation of Diets with Increased Amounts of Dried Coliflower 

The aim of this trial was to assess the *in vitro* ruminal fermentation of diets, including increasing amounts of dried CAU. Five CAU pieces were obtained at local markets, and the whole pieces (including leaves, stems and sprouts) were cut into pieces and dried (40 °C). A control diet containing 40% alfalfa hay and 60% concentrate (fresh matter basis) was formulated to be representative of the diets fed to dairy ruminants in the practice. Three additional diets with the same alfalfa hay:concentrate ratio were formulated by partially replacing feed ingredients in the control concentrate with 8, 16 or 24 g of dried CAU per 100 g of concentrate. All concentrates were formulated to have similar CP and NDF content. All feed ingredients and CAU were ground (1 mm sieve) before being mixed, and a subsample of CAU was taken for chemical analyses.

Samples of the four diets were fermented *in vitro* using the procedure described in Experiment 1 to measure the kinetics of gas production. In addition, 24-h *in vitro* incubations were conducted as described earlier with the exception that incubations were performed in 120-mL vials and the size of sample and buffered rumen fluid was increased to 400 mg DM and 40 mL, respectively, to get enough gas for CH_4_ analyses. After 8 h of incubation, gas production was measured and a sample (about 10 mL) was taken into evacuated tubes for CH_4_ analysis. Immediately, 1 mL of vials content was sampled using a 1-mL plastic syringe, mixed with 1 mL 0.5 M HCl, and frozen (−20 °C) until VFA and NH_3_-N analyses. At the end of the incubation (24 h), the pH of vials content was measured, and samples of gas and vials content were taken for analyses of CH_4,_ VFA and NH_3_-N.

### 2.5. Chemical Analyses, Calculations and Statistical Analyses

The procedures of the Association of Official Analytical Chemists [23] were used to analyze the DM (method 934.01), ash (method 942.05), and EE (method 920.39) content in the tested samples. The N content was determined according to the Dumas method using a TruSpec CN equipment (Leco Corp. St. Joseph, MI, USA). Analysis of NDF was carried out according to Van Soest et al. [24] and that of ADF and lignin according to Robertson and Van Soest [25]. All fiber analyses were carried out using an Ankom 220 Fiber Analyzer unit (Ankom Technology Corp., Macedon, NY, USA), and results were expressed exclusive of residual ash. Sugars were analyzed as described by Marcos et al. [26]. Concentrations of CH_4_ in the gas produced and of VFA in the vials content were analyzed by gas chromatography following the procedures described by García-Martínez et al. [27] and Martínez et al. [28], respectively. Finally, NH_3_-N concentrations were determined by the phenol-hypochlorite method of Wheatherburn [29]. All analyses were performed in duplicate.

The NLIN procedure of SAS [30] was used to fit the gas production values to the exponential model proposed by Krishnamoorthy et al. [31] Gas = A (1 – e ^(–c (t–*lag*))^), in which A is the potential gas production, *c* is the fractional rate of gas production, *lag* is the time before starting gas production, and t is the time of measurement. The rate of gas production between the start of the incubation and the time at which the 50% of potential gas production (AGPR; average gas production rate) was calculated as AGPR = A *c*/[2 (ln2 + *c* × *lag*)] proposed by García-Martínez et al. [27]. The DM effective degradability (DMED) for a rumen digesta passage of 0.042 per h (kp) was estimated as DMED = [(PDDM × *c*)/(*c* + kp)] e ^(−kp × lag)^. The 0.042 per h passage rate corresponds to a digesta rumen retention time of 24 h, which can be found in sheep fed at moderate intake levels [32]. The apparently fermented OM (AFOM) in each vial was estimated from VFA production, as proposed by Demeyer [33]. Finally, the ME content (MJ/kg DM) of the samples was calculated from gas production at 24 h (GP2_24_; mL/200 mg DM sample) and the CP and EE content (expressed in g/kg DM) using the following equation proposed by Menke and Steingass [34]: ME = 2.04 + 0.1448 GP_24_ + 0.0036 CP + 0.0243 EE.

The PROC MIXED of SAS [30] was used to analyze fermentation data from Experiment 1 as a mixed model including the fixed effect of vegetable (CAU and ROM), fraction (leaves, stems and sprouts) and the vegetable × fraction interaction, and the inoculum as a random effect. Data on chemical composition of vegetable fractions were analyzed using the same model but excluding the random effect of the inoculum. The fractions of the three samples of each vegetable (each obtained in a different week) were used as replicates. Data from Experiment 2 were first analyzed as a repeated measure model, but diet × time interactions were detected for several parameters. Therefore, data were analyzed independently for each incubation time (8 and 24 h) as a mixed model, in which the fixed effect was the diet and the random effect was the inoculum. In addition, non-orthogonal polynomial contrasts were performed to test the linear and quadratic effects of CAU inclusion in the diet. In all analyses, the significance level was set at *p* < 0.05, and *p* < 0.10 values were considered as trends. When a significant fixed effect was detected, means were compared with the Tukey’s test. 

## 3. Results and Discussion

### 3.1. Experiment 1. In Vitro Ruminal Fermentation and Intestinal Digestibility of Cauliflower and Romanesco Wastes 

The proportion and chemical composition of each vegetable fraction (leaves, stems and sprouts) in both CAU and ROM are shown in Table 1. Sprouts were the predominant fraction (*p* < 0.05) in CAU accounting for nearly half of the vegetable, whereas leaves were the greatest fraction in ROM. There were vegetable × fraction interactions (*p* = 0.007 to 0.031) for DM, organic matter, CP, NDF, ADF, hemicellulose and the amount of CP insoluble in NDF (NDICP), and a trend for cellulose (*p* = 0.058). As expected, all fractions of both vegetables had low DM content, which ranged from 5.85 to 9.65% and was lower (*p* < 0.001) in CAU than in ROM. Compared with CAU, ROM samples had greater (*p* = 0.004) CP content but lower (*p* ≤ 0.034) sugars, NDF, ADF, hemicellulose and cellulose content. In general, the sprouts of both vegetables had greater CP and EE content and less fiber and lignin content and lignin/NDF ratio than leaves and stems, although the stems and sprouts of ROM had similar (*p* > 0.05) contents of NDF, ADF and cellulose. Our results agree well with the high levels of CP (from 17 to 41% of DM) and low contents of NDF (≤30% of DM) and ADF (≤22% of DM), previously reported for whole CAU [8,10,11] and CAU leaves or mixtures of leaves and stems [9,12,35,36]. All fractions of both CAU and ROM had high sugar content, but especially the stems (about 28% of DM in both vegetables). Wadhwa and Bakshi [35] reported a lower content of total sugars (18.6% of DM) in CAU leaves. As previously discussed by de Evan et al. [20], several factors, such as stage of growth, season, species and variety, soil types, and growth environment, can influence the chemical composition of vegetables, influencing the results obtained in the different studies. To our knowledge, only Lamba et al. [36] have previously reported lignin values in CAU or ROM, and the reported value (3.9% of DM for CAU leaves) is in accordance with the low lignin content (<4.1% on DM basis) observed in our study for all fractions of both vegetables, but especially the sprouts. As a consequence, the lignification of the cell wall (calculated as percentage of lignin in the NDF) was below 9 and 20% in CAU and ROM fractions, respectively. There were differences among fractions in both vegetables. Whereas there were no differences (*p* > 0.05) among CAU fractions in CP and sugars content, ROM sprouts had greater differences (*p* < 0.05) in CP and EE content than leaves and stems, and ROM stems had greater differences (*p* < 0.05) in sugars content compared with leaves and sprouts. Leaves had the greatest (*p* < 0.05) NDF and ADF content in ROM, but in CAU, both leaves and stems had similar (*p* > 0.05) NDF and ADF content.

Table 2 shows the gas production parameters of the analyzed samples. Vegetable × fraction interactions were only detected for the fractional rate of gas production (*p* = 0.016) and DMED (*p* < 0.001). Whereas ROM stems had the greatest (*p* < 0.05) value for both parameters, for CAU there were no differences (*p* > 0.05) among fractions in the fractional rate of gas production and both stems and sprouts had similar DMED (*p* > 0.05). Compared with ROM, CAU fractions have greater (*p* ≤ 0.039) potential gas production (A; 237 vs. 230 mL/g DM), fractional rate of gas production (5.14 vs. 4.86 h) and lag (3.72 vs. 3.31 h), but there were no differences (*p* ≥ 0.119) between vegetables in AGPR, DMED or ME content. Our results for CAU are in good agreement with the high gas production (237–239 mL of gas per g DM) and short lag times reported by others [3,10] after *in vitro* incubation with sheep rumen fluid for 96 h. For both CAU and ROM, stems was the fraction with the greatest (*p* < 0.05) values of A, AGPR and DMED, although there were no differences (*p* > 0.05) between stems and sprouts in DMED for CAU. Leaves had the greatest (*p* < 0.05) lag values and lowest (*p* < 0.05) DMED of the three analyzed fractions, which is consistent with the higher NDF content and lower sugar content of this fraction compared with sprouts and stems.

Both CAU and ROM sprouts had greater (*p* < 0.05) ME content than leaves, with stems having intermediate values, as shown in Table 2. Our values were close to the 10.1 MJ/kg DM reported for a CAU sample by Marino et al. [10] estimated from *in vitro* gas production and chemical composition. Wadhwa et al. [12] obtained a ME content of 13.6 MJ/kg DM in CAU leaves measured in a feeding trial using bucks, but this value was calculated without taking into account the energy lost as CH_4_ because gas emissions were not determined. In contrast, Lamba et al. [36] estimated a lower value (7.73 MJ/kg DM) for a sample of CAU leaves using ruminal fluid from buffalo calves. In our study, one sample of each sugar beet pulp and wheat DDGS was included in the incubations for comparative purposes. The estimated ME content of the sugar beet pulp sample was 9.63 MJ/kg DM, which is only slightly lower than the 9.9 MJ/kg DM value reported by the NRC [37] and the 10. MJ/kg DM assumed by the FEDNA [38]. In contrast, both the NRC [37] and the FEDNA [38] reported greater values for wheat DGGS (12.6 and 11.3 MJ/kg DM, respectively) than the 9.73 MJ/kg DM observed in our study. The underestimation was probably due to the high protein content of DDGS (32.9% of DM), as protein fermentation generates lower amounts of gas than carbohydrate degradation [39,40]. Our results indicate that the DM of stems and sprouts from both CAU and ROM have similar ME content than sugar beet pulp, although the value might be even greater due to the underestimation caused by their high CP content, especially in the sprouts.

The fermentation parameters determined after 24-h of incubation, as shown in Table 3 confirmed that all fractions of CAU and ROM were rapidly and extensively degraded by ruminal microorganisms. There were no vegetable × fraction interactions for any parameter measured, with the exception of final pH (*p* = 0.011). Sprouts had the lowest (*p* < 0.05) pH for CAU, which is consistent with the lower NDF and ADF content of this fraction compared with leaves and stems. In contrast, sprouts in ROM had the greatest (*p* < 0.05) pH for ROM, which might be related to their high sugar content, as numerous studies (reviewed by Oba et al. [41]) reported that rumen pH is not negatively affected by feeding sugars, despite their rapid fermentation. No differences (*p* ≥ 0.162) between the two vegetables were detected in the amount of gas produced, total VFA production, molar proportions of propionate, and acetate/propionate ratio. Values of gas production, final pH and total VFA production for both vegetables were intermediate between those observed for sugar beet pulp and wheat DDGS. The fermentation of ROM resulted in greater proportions (*p* ≤ 0.001) of butyrate and minor VFA (sum of isobutyrate, isovalerate, and valerate) and tended (*p* = 0.090) to lower acetate proportions compared with CAU. The greater proportions of minor VFA in ROM are consistent with both the greater (*p* = 0.008) NH_3_-N concentrations and the greater CP content, as shown in Table 1, as minor VFA are generated in the deamination of branched amino acids and NH_3_-N is one of the major products of CP degradation in the rumen [42]. Concentrations of NH_3_-N in both vegetables were greater than those observed for sugar beet pulp, which is consistent with the high CP content of CAU and ROM. Both ROM sprouts and wheat DGGS had similar CP content (33.0 and 32.9%, respectively), but NH_3_-N concentrations were greater for ROM (380 and 276 mg/L), which was probably be due to the previously reported low CP degradability of wheat DDGS [37,38].

There were marked differences (*p* ≤ 0.017) among vegetable fractions in all 24-h fermentation parameters. For CAU, the production of gas and total VFA was greater for stems and sprouts (*p* = 0.008 and *p* = 0.017, respectively) than for leaves, but stems had the greatest (*p* < 0.05) total VFA in ROM. There were also differences among fractions in VFA profile, with leaves having the greatest (*p* < 0.05) acetate and the lowest (*p* < 0.05) propionate proportion, which is in accordance with the greatest NDF and ADF content in leaves compared with stems and sprouts. These results indicate that the fermentation of leaves was less energetically efficient than that of stems and sprouts [43], which is consistent with the lower ME content estimated for this fraction. Acetate/propionate ratio in stems and sprouts of both vegetables was slightly lower than that observed for sugar beet pulp. 

As shown in Table 4, CAU and ROM had similar (*p* ≥ 0.310) rumen degradability and intestinal digestibility values for both DM and CP, but vegetable × fraction interactions (*p* ≤ 0.019) were detected for DM and CP *in situ* degradability. Leaves have lower (*p* < 0.05) rumen DM degradability than stems and sprouts in CAU, but in ROM the value for sprouts did not differ (*p* > 0.05) from that in leaves and stems. Whereas there were no differences in rumen CP degradability among fractions in CAU, ROM sprouts had lower (*p* < 0.05) values compared to leaves and stems. The rumen degradability of DM measured after 12-h of in *situ* incubation ranged from 78.7 to 91.7% and was lower (*p* < 0.05) for leaves compared with stems and sprouts in both vegetables. Similarly, Wadhwa and Bakshi [35] determined the *in situ* DM rumen degradability of CAU leaves in buffaloes and reported a similar degradability (78.3%) and a high rapidly-soluble fraction (46.7%). Arias et al. [44] also reported a similar value (79.0%) for *in vitro* DM degradability of CAU leaves. In our study, rumen CP degradability ranged from 80.9 to 90.3%, and was greater (*p* < 0.05) for leaves and stems compared with sprouts for ROM, without differences among fractions for CAU. The *in vitro* intestinal digestibility of the by-pass DM was low (<66.0%) for all fractions excepting ROM sprouts that reached 73.3%. In contrast, *in vitro* intestinal digestibility of by-pass CP was high (85.7–93.2%). However, the low CP-bypass fraction of all analyzed samples (9.7–19.1% of total CP content; calculated as 100 minus the amount of CP degraded in the rumen after 12 h of *in situ* incubation) suggests that the intestinal amino acid supply from CAU and ROM would be of limited importance. These results confirm that both CAU and ROM are sources of highly degradable DM and CP.

### 3.2. Experiment 2. In Vitro Fermentation of Diets Including Dried Cauliflower

As there were only small differences in the *in vitro* fermentation of both vegetables, cauliflower was chosen for Experiment 2 because its worldwide production is considerably greater than that of Romanesco [45]. The CAU sample contained 84.7, 27.5, 5.89, 25.5 and 20.9 g of OM, CP, EE, NDF and ADF per 100 of DM, respectively. These data agree well with the composition of the CAU samples used in Experiment 1, with the exception that CP content was slightly greater. Differences in the chemical composition of a vegetable are mainly attributed to stage of growth, season, variety, soil types and growth environment, among others [20]. The control diet was formulated to be representative of those fed to dairy ruminants in the practice, and therefore contained 40% alfalfa hay and 60% high-cereal concentrate (77% of cereals). As intended, all diets had similar CP and NDF contents, as shown in Table 5.

As shown in Table 6, there was a quadratic response (*p* = 0.017) in the potential gas production (A), which increased up from control to CAU diets and decreased for CAU24 diet. A linear decrease (*p* = 0.043) in the lag time was detected as the amount of CAU in the concentrate increased, but there were no differences among diets in other gas production parameters and DMED values. These results are in accordance with the high sugars content of CAU, as sugars are rapidly and extensively fermented in the rumen. A more rapid fermentation of the diets, including CAU compared with the control diet, was also indicated by the linear increase (*p* = 0.015) in total VFA production observed for the CAU-containing diets at 8 h of fermentation, as shown in Table 6. Compared with the control diet, total VFA production in the CAU24 diet was increased by 8.7 and 7.0% at 8 and 24 h incubation, respectively. The linear increase in the amount of AFOM (*p* = 0.018 and 0.026 at 8 and 24 h of incubation, respectively) also supports these results. As VFAs are the main source of energy for ruminants [43], the results indicate that the diets including CAU would supply more energy for the host animal.

The inclusion of CAU in the diet affected molar proportions of the main VFA, as shown in Table 6. At both incubation times, acetate increased (*p* ≤ 0.030; linear) and propionate and butyrate decreased (*p* ≤ 0.042; linear), with the exception of butyrate at 24 h that remained unchanged (*p* = 0.100). As a consequence, acetate/propionate ratios were greater (*p* < 0.05) for CAU16 and CAU24 diets compared with the control one at both incubation times. These results are consistent with the high acetate proportions observed in the fermentation of CAU fractions in Experiment 1. In contrast, the proportions of minor VFA (isobutyrate, valerate and isovalerate) were not affected by the dietary inclusion of CAU. When the production of each VFA at 24 h was calculated by multiplying total VFA production by its molar proportion, the fermentation of all CAU diets resulted in greater (*p* < 0.05) production of acetate (785, 837, 873 and 886 µmol/vial for control, CAU8, CAU16 and CAU24 diets, respectively) without changes in the production of propionate (294, 306, 307 and 305 µmol/vial, respectively) and butyrate (164, 171, 175 and 174 µmol/vial, respectively) production.

The observed trend to a quadratic increase in NH_3_-N concentrations (*p* = 0.057) at 8 h of incubation as the amount of CAU in the diet augmented is in accordance with the high ruminal degradability of CP in CAU, as shown in Table 4. The *in situ* degradability of CP was greater than 85.0% for all CAU fractions, whereas lower degradability values (62–65%) have been reported for soybean meal [37,38], that was the main protein feed in the control concentrate. However, it should be taken into account that ruminal NH_3_-N concentrations are difficult to interpret, as they reflect the balance between the NH_3_-N generated by protein degradation and the NH_3_-N captured by ruminal microorganisms for microbial protein synthesis [46]. Moreover, it should be noted that processes such as NH_3_-N absorption and urea recycling do not occur in the *in vitro* systems, and therefore the direct extrapolation of *in vitro* NH_3_-N concentrations to *in vivo* conditions is not possible.

Including increasing amounts of CAU in the diet tended to increase (*p* = 0.087; quadratic), as shown in Table 6, the CH_4_ production at 8 h of incubation, but this effect disappeared after 24 h. The increased CH_4_ production in CAU-containing diets observed at 8 h was probably due to their greater fermentation, as indicated by the greater VFA production. The lack of differences among diets (*p* ≥ 0.372) in the CH_4_/VFA ratio at 8 h of incubation supports this hypothesis. These results would indicate a lack of antimethanogenic compounds in CAU. In agreement with previous results [47] the proportion of CH_4_ in the gas produced was lower at 8 h (15.9, 16.3, 16.6 and 16.3% for control, CAU8, CAU16 and CAU24 diets, respectively) than at 24 h of fermentation (20.5, 20.6, 20.2 and 20.7%, respectively). These proportions cannot be directly compared with those reported *in vivo*, as some of the CO_2_ produced *in vitro* arises from the incubation medium due to the production of CO_2_ in the neutralization of VFA by bicarbonate [48].

## 4. Conclusions

Leaves, stems and sprouts of cauliflower and Romanesco have high water content, but their dry matter contains high amounts of protein and sugars, and small proportions of low-lignified fiber. All fractions were highly degradable in the rumen (>78% in 12-h *in situ* incubations), and the intestinal digestibility of the small by-pass fraction was low for dry matter (<74%) but high for protein (>85%). Both CAU and ROM are sources of high-degradable dry matter and protein, which can be rapidly fermented by rumen microorganisms. Dried cauliflower can be included up to 24% in a concentrate for dairy sheep without having any negative effect on *in vitro* ruminal fermentation and increasing the amount of organic matter fermented. *In vivo* trials are needed to confirm these results and to assess the potential effects of these vegetables on animal health and product quality.

## Figures and Tables

**Table 1:** Proportion and chemical composition of leaves, stems and sprouts of cauliflower and Romanesco samples (*n* = 3) and chemical composition of sugar beet pulp and wheat DDGS samples used as reference feeds.

Item	Cauliflower			Romanesco			SEM ^1^	*p*			Reference Feeds	
	Leaves	Stems	Sprouts	Leaves	Stems	Sprouts		Vegetable	Fraction	Vegetable × Fraction	Sugar Beet Pulp	Wheat DDGS
Proportion (% of fresh matter)	29.9 ^a^	21.8 ^a^	48.3 ^b^	45.8 ^b^	23.1 ^a^	31.1 ^a^	3.13	0.999	<0.001	<0.001	-	-
Chemical composition ^2^												
Dry matter (%)	6.86	5.85	6.58	6.74 ^a^	7.63 ^a^	9.65 ^b^	0.403	<0.001	0.008	0.007	89.1	92.2
Organic matter	85.4 ^a^	88.3 ^b^	88.0 ^b^	84.6 ^a^	88.2 ^b^	90.6 ^b^	0.56	0.210	<0.001	0.025	94.9	95.5
Crude protein (CP)	21.9	19.9	25.0	21.5 ^a^	23.5 ^a^	33.0 ^b^	1.26	0.004	<0.001	0.020	9.44	32.9
Ether extract	3.62 ^a^	3.91 ^a^	5.50 ^b^	3.08 ^a^	3.37 ^a^	5.47 ^b^	0.278	0.129	<0.001	0.574	0.80	4.61
Sugars	25.5	28.7	23.2	16.3 ^a^	28.3 ^b^	18.9 ^a^	2.36	0.034	0.011	0.220	13.5	6.97
Neutral detergent fiber (NDF)	32.3 ^b^	30.4 ^b^	24.7 ^a^	30.3 ^b^	21.6 ^a^	23.8 ^a^	1.16	0.002	0.001	0.011	48.0	29.5
Hemicellulose	11.9	12.1	10.7	9.68	7.08	11.9	0.83	0.012	0.154	0.009	23.8	18.3
Acid detergent fiber	20.4 ^b^	18.3 ^b^	14.0 ^a^	20.6 ^b^	14.5 ^a^	11.9 ^a^	0.64	0.004	<0.001	0.031	24.2	11.2
Cellulose	18.0 ^b^	15.5 ^b^	13.2 ^a^	17.3 ^b^	10.5 ^a^	11.0 ^a^	0.81	0.002	<0.001	0.058	22.0	7.87
Lignin	2.42 ^b^	2.77 ^b^	0.78 ^a^	3.29 ^b^	4.03 ^b^	0.86 ^a^	0.568	0.137	0.002	0.589	2.16	3.33
Lignin (% of NDF)	7.47	8.98	3.17	10.9 ^b^	19.1 ^c^	3.67 ^a^	2.751	0.060	0.008	0.243	4.49	11.2
NDICP (% CP) ^3^	13.1	20.0	18.2	18.8	10.3	18.4	2.47	0.545	0.427	0.028	NA ^4^	NA ^4^

^a,b,c^ Within each vegetable, means in the same row with different superscript differ (*p* < 0.05); ^1^ SEM: standard error of the mean for the interaction vegetable × fraction (*n* = 3); ^2^ Expressed as g/100 g of dry matter unless otherwise stated; ^3^ NDICP: neutral detergent insoluble crude protein expressed as g/100 g crude protein; ^4^ not analyzed.

**Table 2:** Gas production parameters of different fractions of cauliflower and Romanesco and two reference feeds (sugar beet pulp and wheat DDGS) measured using sheep rumen fluid as inoculum.

Item	Cauliflower			Romanesco			SEM ^2^	*p*			Reference Feeds	
	Leaves	Stems	Sprouts	Leaves	Stems	Sprouts		Vegetable	Fraction	Vegetable × Fraction	Sugar Beet Pulp	Wheat DDGS
A ^1^ (mL/g dry matter)	227 ^a^	252 ^b^	233 ^a^	227 ^a^	242 ^b^	221 ^a^	3.89	0.018	<0.001	0.320	329	185
*c* (%/h)	5.00	5.34	5.07	4.38 ^a^	5.59 ^b^	4.60 ^a^	0.155	0.033	<0.001	0.016	5.21	4.15
lag (h)	4.61 ^b^	3.52 ^a^	3.04 ^a^	4.54 ^b^	2.68 ^a^	2.71 ^a^	0.243	0.039	<0.001	0.284	3.83	0.00
AGPR (mL/h)	6.16 ^a^	7.70 ^b^	6.98 ^b^	5.60 ^a^	8.06 ^b^	6.17 ^a^	0.258	0.119	<0.001	0.065	9.52	2.55
DMED (%)	38.3 ^a^	44.8 ^b^	44.9 ^b^	35.8 ^a^	49.3 ^c^	43.5 ^b^	0.70	0.225	<0.001	< 0.001	38.0	30.1
ME (MJ/kg DM) ^3^	9.03 ^a^	10.0 ^ab^	10.5 ^b^	9.57 ^a^	10.1 ^ab^	11.0 ^b^	0.217	0.830	<0.001	0.930	9.63	9.73

^a,b,c^ Within each vegetable, means in the same row with different superscript differ (*p* < 0.05); ^1^ A: asymptotic (potential) gas production; *c*: fractional rate of gas production; lag: time before starting the gas production; AGPR: average gas production rate; DMED: dry matter effective degradability calculated for a rumen digesta passage of 0.042 per h; ^2^ SEM: standard error of the mean for the interaction vegetable × fraction (*n* = 12); ^3^ ME: metabolizable energy was calculated from gas production at 24 h and chemical composition as proposed by Menke and Steingass [34].

**Table 3:** Fermentation parameters of different fractions of cauliflower and Romanesco and two reference feeds (sugar beet pulp and wheat DDGS) samples (200 mg dry matter) measured in 24-h *in vitro* incubations using sheep rumen fluid as inoculum.

Item ^1^	Cauliflower			Romanesco			SEM ^2^	*p*			Reference Feeds	
	Leaves	Stems	Sprouts	Leaves	Stems	Sprouts		Vegetable	Fraction	Vegetable × Fraction	Sugar Beet Pulp	Wheat DDGS
Gas (mL)	31.5 ^a^	36.5 ^b^	35.0 ^b^	33.6	36.1	34.5	0.49	0.626	0.008	0.330	43.9	26.3
pH	6.67 ^c^	6.58 ^b^	6.54 ^a^	6.60 ^a^	6.61 ^a^	6.73 ^b^	0.011	0.531	0.003	0.011	6.56	6.73
Total volatile fatty acids (VFA; µmol/vial)	1417^a^	1531^b^	1522 ^b^	1487 ^a^	1607 ^b^	1501 ^a^	17.2	0.162	0.017	0.323	1700	1311
Molar proportions of individual VFA (mol/100 mol)												
Acetate (Ac)	66.7 ^b^	62.5 ^a^	61.4 ^a^	65.0 ^b^	61.3 ^a^	60.7 ^a^	0.40	0.090	<0.001	0.836	65.5	53.4
Propionate (Pr)	21.8 ^a^	25.7 ^b^	25.3 ^b^	22.3 ^a^	25.6 ^b^	24.1 ^b^	0.38	0.618	0.001	0.511	25.2	33.3
Butyrate	8.18	8.35	8.48	8.46 ^a^	9.07 ^b^	9.09 ^b^	0.057	<0.001	0.003	0.173	6.93	6.34
Minor VFA	3.29 ^a^	3.42 ^a^	4.74 ^b^	4.22 ^a^	4.03 ^a^	6.13 ^b^	0.144	0.001	<0.001	0.420	2.31	6.96
Ac/Pr (mol/mol)	3.07 ^b^	2.44 ^a^	2.43 ^a^	2.94 ^b^	2.40 ^a^	2.54 ^a^	0.061	0.853	<0.001	0.608	2.60	1.61
NH_3_-N (mg/L)	267	233	288	297 ^a^	293 ^a^	380 ^b^	11.7	0.008	0.028	0.443	108	276

^a,b,c^ Within each vegetable, means in the same row with different superscript differ (*p* < 0.05); ^1^ Each vial contained 200 mg of sample dry matter; Minor VFA: calculated as the sum of isobutyrate, isovalerate, and valerate; ^2^ SEM: standard error of the mean for the interaction vegetable × fraction (*n* = 12).

**Table 4:** Rumen degradability after 12 h of in *situ* incubation and *in vitro* intestinal digestibility of dry matter (DM) and crude protein (CP) of different fractions of cauliflower and Romanesco.

Item	Cauliflower			Romanesco			SEM ^1^	*p*		
	Leaves	Stems	Sprouts	Leaves	Stems	Sprouts		Vegetable	Fraction	Vegetable × Fraction
*In situ* rumen degradability (%)										
DM	78.7 ^a^	88.1 ^b^	91.4 ^b^	84.0 ^a^	91.7 ^b^	86.4 ^a^	1.60	0.310	<0.001	0.019
CP	85.5	87.9	88.5	87.3 ^b^	90.3 ^b^	80.9 ^a^	1.64	0.421	0.032	0.005
*In vitro* intestinal digestibility (%)										
DM	54.8	65.8	64.4	52.6 ^a^	57.1 ^a^	73.3 ^b^	2.44	0.859	0.022	0.170
CP	85.7	91.1	90.2	86.5 ^a^	88.9 ^ab^	93.2 ^b^	1.01	0.727	0.001	0.375

^a,b,c^ Within each vegetable, means in the same row with different superscript differ (*p* < 0.05); ^1^ SEM: standard error of the mean (*n* = 9).

**Table 5:** Concentrate ingredients and the chemical composition of experimental diets containing increased amounts of cauliflower (CAU) used in Experiment 2 ^1^.

Item	Diet			
	Control	CAU8	CAU16	CAU24
Concentrate ingredients (g/100 g fresh matter)				
Dried cauliflower	-	8.0	16.0	24.0
Corn	32.0	32.0	32.0	32.0
Barley	30.0	30.0	30.0	30.0
Wheat	15.0	11.0	7.5	4.0
Soybean meal 46%	14.0	12.0	10.0	8.0
Wheat bran	7.0	5.0	2.5	0.0
Others ^2^	2.0	2.0	2.0	2.0
Chemical composition (g/100 g dry matter unless otherwise stated) ^3^				
Dry matter (g/100 g)	89.7	89.7	89.7	89.7
Organic matter	93.0	92.2	91.2	90.3
Crude protein	16.1	16.1	16.1	16.1
Ether extract	4.18	4.28	4.37	4.46
Neutral detergent fiber	31.5	31.7	31.7	31.8
Acid detergent fiber	15.9	16.4	17.0	17.6

^1^ All diets were composed of 40% alfalfa hay and 60% concentrate (fresh matter basis); ^2^ All diets contained calcium soap, calcium carbonate and mineral/vitamin premix in proportions of 1.0, 0.5 and 0.5%, respectively (fresh matter basis); ^3^ Chemical composition of diets was calculated from the analyzed composition of each feed ingredient.

**Table 6:** Gas production and fermentation parameters of experimental diets containing 40% alfalafa hay and 60% concentrate with increased amounts of Brussels sprouts (BS; 8, 16 and 24% of concentrate fresh matter).

Item	Diet				SEM ^2^	*p*	
	Control	CAU8	CAU16	CAU24		Lineal	Quadratic
**Gas production parameters ^1^**							
A (mL/g DM)	280 ^a^	287 ^b^	290 ^b^	283 ^a^	2.50	0.376	0.017
*c* (%/h)	3.90	3.80	3.39	3.90	0.001	0.493	0.514
lag (h)	1.10 ^b^	0.89 ^ab^	0.91 ^ab^	0.76 ^a^	0.09	0.043	0.725
AGPR (mL/h)	7.40	7.58	7.77	7.75	0.19	0.195	0.616
DMED (%)	40.5	40.9	41.3	41.7	0.48	0.100	0.900
**Fermentation parameters (8 h)**							
Total volatile fatty acids (VFA; µmol)	1284 ^a^	1354 ^ab^	1396 ^b^	1406 ^b^	30.4	0.015	0.340
Individual VFA (mol/100 mol)							
Acetate (Ac)	61.1 ^a^	61.8 ^b^	62.5 ^c^	63.0 ^c^	0.18	<0.001	0.759
Propionate (Pr)	22.9 ^b^	22.6 ^b^	22.0 ^ab^	21.7 ^a^	0.18	<0.001	0.797
Butyrate	12.8 ^b^	12.6 ^b^	12.5 ^ab^	12.3 ^a^	0.11	0.004	0.876
Minor VFA ^3^	3.11	2.99	3.05	2.98	0.08	0.408	0.742
Ac/Pr (mol/mol)	2.69 ^a^	2.77 ^a^	2.87 ^b^	2.92 ^b^	0.03	<0.001	0.721
NH_3_-N (mg/L)	143	157	149	141	5.1	0.581	0.057
CH_4_ (mL)	6.90	7.41	7.68	7.28	0.24	0.213	0.087
CH_4_/VFA (mL/mmol)	5.40	5.52	5.52	5.21	0.23	0.577	0.372
AFOM (mg/vial) ^4^	114 ^a^	120 ^ab^	124 ^b^	125 ^b^	2.69	0.018	0.323
**Fermentation parameters (24 h)**							
pH	6.79	6.79	6.79	6.78	0.01	0.827	0.745
Total VFA (µmol)	2446 ^a^	2603 ^b^	2618 ^b^	2631 ^b^	25.7	<0.001	0.045
Individual VFA (mol/100 mol)							
Acetate (Ac)	61.5 ^a^	61.9 ^ab^	62.5 ^b^	62.8 ^b^	0.29	0.030	0.598
Propionate (Pr)	18.7 ^b^	18.7 ^b^	18.3 ^ab^	17.9 ^a^	0.16	0.042	0.712
Butyrate	15.5	15.2	15.0	15.1	0.18	0.100	0.414
Minor VFA ^3^	4.27	4.15	4.16	4.24	0.08	0.828	0.308
Ac/Pr (mol/mol)	3.31 ^a^	3.35 ^ab^	3.43 ^b^	3.53 ^b^	0.04	0.021	0.843
NH_3_-N (mg/L)	189	199	198	205	4.5	0.142	0.332
CH_4_ (mL)	14.9	15.6	15.0	15.4	0.36	0.793	0.513
CH_4_/VFA (mL/mmol)	6.10	5.99	5.74	5.85	0.12	0.105	0.479
AFOM (mg/vial) ^4^	220 ^a^	234 ^b^	235 ^b^	236 ^b^	2.35	0.026	0.264

^a,b,c^ Means in the same row with different superscript differ (*p* < 0.05). ^1^ Determined in 144-h *in vitro* incubations with ruminal fluid from sheep; A: potential gas production; *c*: fractional rate of gas production, lag: is the time needed to start gas production; AGPR: average gas production rate. DMED: dry matter effective degradability estimated for a rumen particulate outflow 0.042 per h. ^2^ SEM: standard error of the mean. **^3^** Minor VFA: calculated as the sum of isobutyrate, isovalerate and valerate. ^4^ AFOM: fermented organic matter estimated from VFA production [28].
